# Gender Differences in Quit Rates in a Tobacco Cessation Program: In Search of Demographic, Socioeconomic, Health, or Behavioral Explanatory Mechanisms

**DOI:** 10.31586/jbls.2025.6017

**Published:** 2025-03-09

**Authors:** Payam Sheikhattari, Rifath Ara Alam Barsha, Shervin Assari

**Affiliations:** 1 Prevention Sciences Research Center, Morgan State University, Baltimore, MD, USA; 2 School of Community Health & Policy, Morgan State University, Baltimore, MD, USA; 3 Johns Hopkins University, Baltimore, MD, USA; 4 University of Mississippi Medical Center, Jackson, MS, USA; 5 Charles R. Drew University of Medicine and Science, Los Angeles, CA, USA

**Keywords:** Gender Differences, Smoking Cessation, Tobacco Use, Intervention Effectiveness, Quit Rates

## Abstract

**Background:**

Women have consistently shown lower quit rates in tobacco cessation programs compared to men. This gender disparity persists despite comprehensive interventions and access to cessation resources. While prior studies suggest that factors such as social support, chronic disease burden, and socioeconomic status may contribute to these differences, there is limited empirical evidence to confirm these mechanisms.

**Aims:**

This study aimed to investigate potential mechanisms underlying gender differences in quit rates in a tobacco cessation program, testing whether demographic, socioeconomic, health, or behavioral factors explain the observed disparities.

**Methods:**

Participants were assigned to one of three smoking cessation interventions: an in-person program (CEASE), a self-help approach, or an online/hybrid program. The main outcome measured was smoking abstinence, evaluated three months after the intervention. Secondary analyses explored whether demographic, socioeconomic, health, or tobacco use-related factors mediated the association between gender and quit rates.

**Results:**

Women had significantly lower quit rates than men (p < 0.01). This association remained significant after adjusting for demographic, socioeconomic, health, and addiction-related factors. While women reported higher social support and a higher prevalence of chronic cardiometabolic conditions, these factors did not explain the gender disparity in quit rates.

**Conclusions:**

Gender differences in quit rates persist despite controlling for known factors that could influence cessation success. Although women had higher social support, they had lower quit rate. Future research should explore unmeasured variables, such as psychological, biological, and structural influences, to develop more effective cessation strategies tailored for women.

## 1. Introduction

Gender differences in smoking cessation have been widely observed [1], with many studies suggesting that men and women may have different quit rates [2]. However, the reasons for these disparities remain complex and multifaceted, involving biological, psychological, social, and structural factors [3]. Some research suggests that men and women may differ in their emotional and psychological reliance on smoking, their experiences of stress, and how they metabolize nicotine [4–6]. Additionally, chronic health conditions, particularly non-fatal cardiometabolic diseases, which are more prevalent among women [7,8], could influence cessation outcomes in ways that are not yet fully understood [9].

Despite these insights, relatively few studies have systematically explored whether these factors fully explain gender differences in quit rates. It is likely that a broad range of interrelated social, health, and behavioral factors contribute to this disparity. For example, higher socioeconomic status (SES) has been linked to greater success in smoking cessation, possibly by providing financial and psychological resources that support quitting [10–12]. At the same time, chronic conditions, while sometimes motivating individuals to quit due to heightened health concerns, could also act as barriers, particularly when smoking is used as a coping mechanism [9]. Depression, which is more common among women, may further complicate cessation efforts by reducing motivation to quit [13–15]. Social support, another key factor in smoking cessation, differs by gender; while women often report stronger social networks, the extent to which these networks facilitate quitting remains unclear [16,17].

It is also important to consider the interplay between gender-related (social) [2]and sex-related (biological) [18] influences on smoking cessation. Some disparities may be shaped by structural and social determinants, such as gender norms [19], caregiving responsibilities, and economic constraints, while others may be linked to biological factors, including hormonal differences and nicotine metabolism [18]. A better understanding of these interconnected influences could help refine interventions that address both social and biological barriers to cessation.

Healthcare utilization and adherence to treatment recommendations represent another relevant dimension. Women are generally more engaged with healthcare services and may be more likely to follow medical advice compared to men [20,21]. In the context of a behavioral intervention such as CEASE [22,23], this pattern raises the possibility that women could demonstrate higher responsiveness due to their greater engagement with healthcare systems. However, the extent to which this translates into better cessation outcomes remains an open question.

Another phenomenon that has been noted in smoking research is the “telescoping effect,” [24–29] which describes how certain populations, including women, may progress more rapidly from initiation to dependence [25–27]. Although women tend to have lower overall smoking prevalence than men, those who do start smoking may experience a faster transition to heavier use and greater difficulty quitting. Similar patterns have been documented among other populations, such as racial and ethnic minorities, suggesting that social and structural factors may play a role in amplifying risks associated with substance use.

This study seeks to explore whether demographic, socioeconomic, health, or behavioral factors help explain gender differences in smoking cessation outcomes within a community-based randomized trial. By investigating these potential mechanisms, this research aims to contribute to the development of more tailored interventions that better address the challenges women face in their efforts to quit smoking.

## 2. Methods

### 2.1. Design and Setting

This study utilized a randomized cluster trial design to evaluate smoking cessation interventions across three Baltimore City communities[23]. Communities were randomly assigned to one of three intervention arms: (1) in-person intervention, (2) virtual/hybrid intervention, and (3) self-help/control group. Participants were recruited from community spaces such as public housing sites, churches, and senior residences.

### 2.2. Ethical Considerations

The study was approved by Morgan State University’s Institutional Review Board (IRB #19/06–0082). Written informed consent was obtained from all participants prior to enrollment.

### 2.3. Study Participants

To be eligible for the study, individuals had to be at least 21 years old, smoke a minimum of three cigarettes per day, express a willingness to quit, and provide informed consent. Those participating in the virtual intervention also needed a suitable device and a reliable internet connection.

### 2.4. Intervention

Nine peer motivators, trained in tobacco cessation, facilitated smoking cessation classes. Sessions followed a structured timeline over seven weeks, covering motivation, quitting strategies, and relapse prevention. In-person participants received printed materials, while virtual participants accessed an online platform with the same content.

### 2.5. Questionnaires

Baseline surveys collected data on demographics, smoking history, physical and mental health, and social support. Follow-up surveys assessed smoking status three months post-intervention.

### 2.6. Measures

**Outcome Variable:** Smoking cessation at follow-up was self-reported (0 = did not quit, 1 = quit).
**Explanatory Variables:** Age, education, income, social support, number of chronic health conditions, nicotine dependence, and depression [23,38,39].
**Independent Variable:** Gender was self-reported as male (coded as 0) or female (coded as 1).

### 2.7 Statistical Analysis

Descriptive statistics were computed for all variables. Bivariate analyses assessed differences by gender, and logistic regression models examined predictors of quitting. We also ran gender-specific logistic regression models. All analyses were conducted using Stata 15.0, with statistical significance set at p < 0.05.

## 3. Results

Table 1 presents the descriptive results of the study, both overall and stratified by gender. Among the 232 participants, the majority were women (50.5%), Black Americans (82.8%), and over 50 years old (78.4%). The majority of participants had lower educational attainment, with only 12.1% reporting a bachelor’s degree or higher.

Among men, 25.2% reported quitting smoking, while 16.2% of women reported doing so. Among the study intervention groups, the percentage of men was highest in the self-help group (39.3%). On the other hand, 45.3% of the participants in the virtual/hybrid group were women. Similar percentages of men and women did not graduate from high school (28.0% and 29.1%, respectively). The mean (SD) score of nicotine dependence at baseline was 4.6 (2.1) for men and 4.3 (2.0) for women. The mean (SD) score of perceived social support was 3.9 (1.1) for men and 4.2 (0.9) for women.

Bivariate correlations among the study variables are presented in Table 2. Perceived social support (r = 0.17, p < 0.05) showed a significant positive correlation with gender. No other variables were significantly correlated with gender.

Table 3 shows the results of the multivariable logistic regression. There are significantly higher odds of quitting (AOR = 3.97, *p* < 0.05) among individuals who received in-person intervention. Women were less likely to quit compared to men (AOR = 0.27, *p* < 0.01). Participants with a higher number of cardiometabolic risk conditions had higher odds of quit smoking (AOR = 1.75, *p* < 0.05), while more depressive symptoms were associated with lower odds of quitting (AOR = 0.60, *p* < 0.05). Menthol/multiple flavor tobacco users were significantly less likely to quit smoking compared to non-users (AOR = 0.19, *p* < 0.01).

Table 4 presents the multivariable logistic regression results, including an interaction term between study arm and gender. The in-person group had a higher likelihood of quitting smoking (AOR = 3.64); however, the result was not statistically significant. The interaction term for the in-person group and female had an AOR of 0.24, but it was also not significant. The interaction terms for the other two study groups did not generate results due to insufficient observations.

Having more cardiometabolic risk conditions was significantly associated with higher odds of quitting smoking (AOR = 1.91, *p* < 0.01), whereas depression was significantly associated with lower odds of quitting smoking (AOR = 0.55, *p* < 0.01).

Table 5 shows the results of the multivariable logistic regression stratified by gender. For men, more depressive symptoms were associated with lower odds of quitting (AOR = 0.24, p < 0.01). For women, no significant association was found between the study variables and quitting smoking.

## 4. Discussion

This study examined whether demographic, socioeconomic, health, or behavioral factors explained gender disparities in quit rates among participants in a community- based tobacco cessation program. Despite testing multiple potential mediators, none fully accounted for the lower quit rates observed among women. This suggests that additional, unmeasured factors may play a critical role in shaping cessation outcomes.

One of the key findings was that, although women had higher levels of social support than men, this did not translate into higher cessation success. Prior studies suggest that social support may function differently for women, with emotional support potentially reinforcing smoking behavior rather than facilitating quitting [16,17,30–33]. Some evidence suggests that women may be more likely to engage in smoking as a coping mechanism for stress, and if their support networks normalize or even enable smoking as a stress-relief strategy, cessation efforts may be undermined.

Additionally, women in our study had a higher prevalence of chronic cardiometabolic conditions, but this factor alone did not explain the gender disparity in quit rates. While chronic disease burden can sometimes serve as a motivator for smoking cessation due to heightened health concerns [9], it may also act as a barrier by increasing psychological distress, which could, in turn, reinforce smoking behavior. The lack of a clear relationship between chronic disease and cessation success suggests that the intersection of health status and smoking behavior is complex and likely influenced by additional factors, such as healthcare access, stress, and coping mechanisms.

Our findings suggest that unmeasured biological, psychological, and structural factors may contribute to lower quit rates among women. Some research has suggested that men and women may differ in experiencing withdrawal symptoms, responsiveness to nicotine replacement therapy, and susceptibility to stress-induced relapse [34–36]. Additionally, structural barriers, including disparities in healthcare access, provider biases, and competing caregiving responsibilities, may further impede cessation success. These findings underscore the importance of considering both biological and social determinants when designing tobacco cessation interventions for women.

While no single factor fully explains the gender differences in smoking cessation, several predictors of cessation emerged in this sample. In bivariate analysis, depression and menthol/multiple flavor use were associated with lower quit rates. In the multivariable model, in addition to menthol use, depression, being female and having lower number of chronic medical conditions were predictors of lower odds of successful cessation, independent of intervention arm. These findings, also discussed here [37–39], align with existing literature, which suggests that menthol may enhance nicotine dependence and increase the difficulty of quitting [40–49]. Menthol’s cooling effect can make smoking more appealing [50–52]. Biological studies have shown that menthol use may lead to higher addiction severity [40–49]. Additionally, research indicates that individuals with multiple chronic medical conditions may be more motivated to quit due to increased health concerns and interactions with healthcare providers [53]. Overall, these results highlight the importance of considering gender, mental health, flavor use, and chronic health conditions when developing targeted smoking cessation interventions. Addressing depression in smoking cessation programs and offering tailored support for individuals who use menthol cigarettes may improve quit rates, particularly among women and those with multiple health conditions.

### 4.1. Implications

These findings highlight the need for gender-sensitive smoking cessation interventions. Standard approaches may be insufficient for addressing the unique challenges faced by women, requiring a more tailored strategy that incorporates behavioral counseling, stress management techniques, and alternative pharmacological interventions that account for sex-based differences in nicotine metabolism and withdrawal severity.

Healthcare providers should be aware of the lower cessation success rates among women and implement gender-specific strategies, such as extended counseling sessions, integration of mental health support, and resources for managing stress-related triggers. Additionally, higher nicotine replacement therapy (NRT) dosages or alternative cessation medications may be necessary for women, as their unique physiological responses to nicotine may require adjustments in pharmacological interventions.

Policy implications include the need for tobacco control strategies that recognize and address gender disparities. Efforts should focus on ensuring equitable access to cessation resources, addressing structural barriers that disproportionately affect women, and developing policies that integrate cessation interventions into broader women’s health initiatives.

It is also important to consider contextual factors that may have influenced the findings. The study was conducted in Baltimore, where high rates of poverty and structural inequalities may have compounded the challenges faced by participants. Additionally, the sample primarily consisted of low-income, ethnically diverse populations, particularly Black women, who may experience distinct barriers to smoking cessation due to systemic disadvantages, economic constraints, and social stressors. These factors are consistent with the intersectionality framework, which posits that the experiences of individuals with multiple marginalized identities cannot be understood by simply summing the effects of each identity. In other words, the smoking cessation experiences of minority women are shaped by a complex interplay of gender, race, socioeconomic status, and other contextual factors that collectively contribute to disparities in quit rates.

Thus, while our findings indicate that women in this program had lower cessation success, we do not conclude that women universally respond less effectively to behavioral interventions. The interaction between gender, socioeconomic status, and racial/ethnic identity must be considered in designing effective cessation strategies.

### 4.2. Limitations

This study has several limitations that should be acknowledged: short follow-up period, unmeasured factors, sample size constraints, and potential measurement gaps. The study only tracked participants for three months post-intervention. A longer follow- up period may have provided greater insight into long-term cessation patterns and relapse rates. Although we examined multiple potential mediators, other key variables— such as hormonal influences, stress-coping mechanisms, and histories of substance use— were not included. These factors could provide a more comprehensive understanding of gender disparities in quit rates. The study’s modest sample size limited statistical power, particularly in testing interaction effects between intervention type and gender. Future studies with larger sample sizes could more effectively explore these relationships. Variables such as past quit attempts, severity of nicotine dependence, and concurrent use of other substances (e.g., alcohol, marijuana) were not fully assessed. These factors may further explain variations in cessation success. Additionally, we cannot rule out the possibility that self-report bias is more pronounced among men. Men may rely on estimation, while women may count more precisely[54,55], potentially engaging different cognitive processes when reporting self-rated quitting. Despite these limitations, the study contributes to a growing body of evidence indicating that gender disparities in smoking cessation are driven by complex and multifactorial influences that extend beyond traditional demographic and socioeconomic explanations.

### 4.3. Future Research Directions

Future research should explore gender-specific barriers and facilitators of smoking cessation in greater depth. Both social and biological determinants—including gender roles, resource availability, caregiving responsibilities, and hormonal fluctuations— should be examined as potential moderators of intervention effectiveness. One key area for future exploration is the role of motherhood and employment-related stress in smoking cessation. Women who balance caregiving responsibilities and employment demands may find it more difficult to engage fully with cessation programs, requiring interventions that specifically address these constraints. Additionally, biological mechanisms, including hormonal fluctuations across the menstrual cycle, pregnancy- related changes, and postmenopausal differences in nicotine metabolism, warrant further investigation. Understanding these biological factors could help tailor interventions more effectively for different life stages. More research is needed to examine the validity of self- reports across genders. It is unknown how sex and gender difference in cognitive style and social desirability may alter the responses of men and women to a question such a self-rated quit. Another promising area for future research is the effectiveness of alternative cessation approaches for women, such as 1) mindfulness-based interventions for stress reduction, 2) combination therapy approaches that integrate pharmacotherapy with intensive counseling, 3) community-based peer support models tailored to women’s needs, and 4) cessation programs embedded within maternal health or primary care settings. Lastly, intersectionality should be central to future research efforts. Given that low-income Black women may face unique challenges in cessation due to systemic inequities, future studies should explicitly examine how race, class, and gender interact to shape smoking behavior and intervention effectiveness.

## 5. Conclusion

Women in this smoking cessation program exhibited lower quit rates than men, and this disparity was not fully explained by demographic, socioeconomic, mental health, physical health, or tobacco-related variables. The persistence of this effect, even after adjusting for multiple potential mediators, suggests that additional mechanisms—such as nicotine metabolism, withdrawal severity, stress-related relapse, and structural barriers— may be at play. Importantly, our findings do not imply that women universally respond worse to behavioral interventions. Instead, the observed disparities likely reflect a combination of biological, psychological, and structural challenges that differentially affect women’s ability to quit smoking. The social context of the study population, including low-income, minority women in an urban setting, may further contribute to the observed gender differences. Further research is needed to uncover the underlying mechanisms driving gender disparities in smoking cessation and to develop more tailored, gender-responsive interventions. By integrating insights from both social and biological determinants, future cessation programs can be better designed to support women in overcoming the unique barriers they face in quitting smoking.

## Figures and Tables

**Table 1.** Study Sample - Overall and by Gender

Variables	Gender			Overall (n=232)
	Men (n=107)	Women (n=117)	Missing (n=8)	
Quit Smoking				
No	80 (74.8)	98 (83.8)	8 (100.0)	186 (80.17)
Yes	27 (25.2)	19 (16.2)	-	46 (19.83)
Study Arm**				
In-person	30 (28.0)	45 (38.5)	4 (50.0)	79 (34.0)
Virtual/Hybrid	35 (32.7)	53 (45.3)	2 (25.0)	90 (38.8)
Self-help	42 (39.3)	19 (16.2)	2 (25.0)	63 (27.2)
Age (years)***				
50 years or less	24 (22.4)	21 (18.0)	2 (25.0)	47 (20.3)
More than 50 years	83 (77.6)	96 (82.0)	3 (37.5)	182 (78.4)
Missing	-	-	3 (37.5)	3 (1.3)
Race***				
Black American	85 (79.4)	104 (88.9)	3 (37.5)	192 (82.8)
White	16 (15.0)	10 (8.6)	1 (12.5)	27 (11.6)
Other/Multiple	4 (3.7)	3 (2.6)	1 (12.5)	8 (3.4)
Missing	2 (1.9)	-	3 (37.5)	5 (2.2)
Educational Attainment***				
Some high school or less	30 (28.0)	34 (29.1)	-	64 (27.6)
Graduated from high school/GED	42 (39.3)	38 (32.5)	2 (25.0)	82 (35.3)
Some college	23 (21.5)	28 (23.9)	3 (37.5)	54 (23.3)
Bachelor or more	11 (10.3)	17 (14.5)	-	28 (12.1)
Missing	1 (0.9)	-	3 (37.5)	4 (1.7)
Other Tobacco Product Use**				
No	80 (74.8)	96 (82.1)	4 (50.0)	180 (77.6)
Yes	25 (23.3)	20 (17.1)	4 (50.0)	49 (21.1)
Missing	2 (1.9)	1 (0.8)	-	3 (1.3)
Menthol/multiple Flavor Use				
No	6 (5.6)	10 (8.5)	2 (25.0)	18 (7.8)
Yes	96 (89.7)	104 (88.9)	6 (75.0)	206 (88.8)
Missing	5 (4.7)	3 (2.6)	-	8 (3.4)
	Mean (SD)	Mean (SD)		Mean (SD)
Nicotine Addiction (at Baseline)	4.6 (2.1)	4.3 (2.0)	-	4.4 (2.0)
Number of Cardiometabolic Risk Conditions	1.0 (0.9)	1.2 (1.0)	-	1.1 (1.0)
Depression	1.2 (1.6)	1.3 (1.7)	-	1.3 (1.7)
Perceived Stress	5.3 (3.0)	5.3 (3.1)	-	5.3 (3.0)
Perceived Social Support*	3.9 (1.1)	4.2 (0.9)	-	4.1 (1.0)

** P < 0.01

**Table 2.** Correlation Matrix of the Study Variables

Variables	1	2	3	4	5	6	7	8	9	10	11
1. Gender (Female)	1.00										
2.Quit Smoking (Yes)	−0.11	1.00									
3.Age	0.06	0.04	1.00								
4. Education	0.05	0.09	−0.01	1.00							
5. Number of Cardiometabolic Risk Conditions	0.12	0.10	0.28***	0.05	1.00						
6. Depression	0.02	−0.14*	0.00	−0.03	0.17*	1.00					
7.Perceived Stress	−0.01	−0.01	−0.17*	−0.02	−0.09	0.42***	1.00				
8. Perceived Social Support	0.17*	0.12	0.14*	0.01	−0.01	−0.31***	−0.33***	1.00			
9.Oher Tobacco Products Use (Yes)	−0.08	0.08	−0.17*	−0.01	−0.09	0.11	0.12	−0.13	1.00		
10. Menthol/Multiple Flavor Use (Yes)	−0.06	−0.14*	0.06	−0.13	0.10	−0.04	−0.02	−0.03	−0.17*	1.00	
11.Nicotine Addiction (at Baseline)	−0.08	−0.08	−0.01	−0.18**	−0.01	0.22***	0.19**	−0.16*	−0.02	0.00	1.00

* P < 0.05;

** P < 0.01;

*** P < 0.001.

**Table 3.** Logistic Regression Results

Variables	Quit Smoking	
	Unadjusted OR (95% CI)	Adjusted OR (95% CI)
Study Arm		
Self-help group	Ref.	Ref.
In-person group	2.17 (0.92, 5.16)	3.97* (1.22, 12.94)
Virtual/hybrid group	1.30 (0.53, 3.16)	2.25 (0.67, 7.53)
Gender		
Men	Ref.	Ref.
Women	0.57 (0.30, 1.11)	0.27** (0.11, 0.65)
Age		
50 years or less	Ref.	Ref.
More than 50 years	1.29 (0.56, 2.98)	1.72 (0.57, 5.21)
Educational Attainment		
Some high school or less	Ref.	Ref.
Graduated from high school/GED	1.08 (0.46, 2.54)	0.78 (0.25, 2.42)
Some college	1.38 (0.55, 3.43)	1.24 (0.37, 4.17)
Bachelor or more	1.93 (0.68, 5.49)	1.16 (0.28, 4.89)
Number of Cardiometabolic Risk Conditions	1.29 (0.91, 1.84)	1.75* (1.12, 2.74)
Depression	0.77* (0.61, 0.98)	0.60** (0.41, 0.88)
Perceived Stress	1.0 (0.90, 1.11)	1.04 (0.88, 1.22)
Perceived Social Support	1.39 (0.96, 2.01)	1.63 (0.96, 2.75)
Other Tobacco Product Use		
No	Ref.	Ref.
Yes	1.61 (0.77, 3.36)	2.46 (0.92, 6.61)
Menthol/Multiple Flavor Use		
No	Ref.	Ref.
Yes	0.36* (0.13, 0.98)	0.19* (0.05, 0.78)
Nicotine Addiction (at Baseline)	0.93 (0.79, 1.09)	1.04 (0.85, 1.29)

Abbreviations: 0R= Odds Ratio; CI=Confidence Interval;

** P < 0.01,

* P < 0.05.

**Table 4.** Logistic Regression Results with Interaction between Study Arms and Gender

Variables	Quit Smoking	
	Unadjusted OR (95% CI)	Adjusted OR (95% CI)
Study Arm		
Self-help group	Ref.	Ref.
In-person group	2.17 (0.92, 5.16)	3.64 (0.91, 14.52)
Virtual/hybrid group	1.30 (0.53, 3.16)	0.88 (0.19, 4.00)
Gender		
Men	Ref.	Ref.
Women	0.57 (0.30, 1.11)	0.76 (0.18, 3.23)
Study arm*Gender		
Self-help* Female	NA	Empty
In-person*Female	NA	0.24 (0.03, 1.76)
Virtual/hybrid* Female	NA	Omitted
Age		
50 years or less	Ref.	Ref.
More than 50 years	1.29 (0.56, 2.98)	1.82 (0.59, 5.67)
Educational Attainment		
Some high school or less	Ref.	Ref.
Graduated from high school/GED	1.08 (0.46, 2.54)	0.93 (0.30, 2.91)
Some college	1.38 (0.55, 3.43)	1.26 (0.37, 4.34)
Bachelor or more	1.93 (0.68, 5.49)	1.01 (0.23, 4.47)
Number of Cardiometabolic Risk Conditions	1.29 (0.91, 1.84)	1.91** (1.19, 3.07)
Depression	0.77* (0.61, 0.98)	0.55** (0.37, 0.83)
Perceived Stress	1.0 (0.90, 1.11)	1.07 (0.90, 1.27)
Perceived Social Support	1.39 (0.96, 2.01)	1.63 (0.95, 2.78)
Other Tobacco Product Use		
No	Ref.	Ref.
Yes	1.61 (0.77, 3.36)	2.55 (0.92, 7.04)
Menthol/Multiple Flavor Use		
No	Ref.	Ref.
Yes	0.36* (0.13, 0.98)	0.13* (0.03, 0.63)
Nicotine Addiction (at Baseline)	0.93 (0.79, 1.09)	1.10 (0.88, 1.36)

Abbreviations: 0R= Odds Ratio; CI=Confidence Interval;

** P < 0.01,

* P < 0.05.

**Table 5.** Stratified Models by Gender

Variables	Quit Smoking			
	Men		Women	
	Unadjusted OR (95% CI)	Adjusted OR (95% CI)	Unadjusted OR (95% CI)	Adjusted OR (95% CI)
Study Arm				
Self-help group	Ref.	Ref.	Empty	Empty
In-person group	2.44 (0.87, 6.90)	2.00 (0.39, 10.20)	1.08 (0.39, 2.93)	0.76 (0.18, 3.23)
Virtual/hybrid group	0.76 (0.24, 2.39)	0.61 (0.11, 3.45)	Omitted	Omitted
Age				
50 years or less	Ref.	Ref.	Ref.	Empty
More than 50 years	0.77 (0.28, 2.12)	1.15 (0.27, 4.96)	4.62 (0.58, 36.68)	Omitted
Educational Attainment				
Some high school or less	Ref.	Ref.	Ref.	Ref.
Graduated from high school/GED	0.55 (0.19, 1.57)	0.37 (0.07, 1.98)	6.19 (0.70, 54.31)	4.80 (0.40, 57.25)
Some college	0.71 (0.21, 2.35)	0.69 (0.10, 4.52)	9.00* (1.01, 79.99)	5.24 (0.43, 63.79)
Bachelor or more	0.44 (0.08, 2.46)	Empty	18.00* (1.95, 166.44)	8.33 (0.58, 120.24)
Number of Cardiometabolic Risk Conditions	1.07 (0.67, 1.72)	1.58 (0.78, 3.21)	1.43 (0.87, 2.34)	2.22 (0.88, 5.61)
Depression	0.51** (0.32, 0.82)	0.24** (0.09, 0.63)	1.01 (0.76, 1.34)	0.77 (0.45, 1.31)
Perceived Stress	0.92 (0.79, 1.06)	1.05 (0.80, 1.40)	1.08 (0.91, 1.27)	1.11 (0.85, 1.46)
Perceived Social Support	1.66* (1.03, 2.67)	1.51 (0.73, 3.13)	1.24 (0.67, 2.29)	2.43 (0.81, 7.31)
Other Tobacco Product Use				
No	Ref.	Ref.	Ref.	Ref.
Yes	1.17 (0.43, 3.20)	2.34 (0.52, 10.41)	2.74 (0.89, 8.39)	3.99 (0.36, 43.66)
Menthol/Multiple Flavor Use				
No	Ref.	Ref.	Ref.	Ref.
Yes	0.67 (0.11, 3.87)	0.06 (0.00, 1.21)	0.16* (0.04, 0.61)	0.08 (0.00, 1.94)
Nicotine Addiction (at Baseline)	0.86 (0.69, 1.06)	1.07 (0.77, 1.51)	0.99 (0.77, 1.27)	1.10 (0.75, 1.62)

Abbreviations: 0R= Odds Ratio; CI=Confidence Interval;

** P < 0.01,

* P < 0.05.
